# Immune modulation for β-cell replacement in type 1 diabetes

**DOI:** 10.3389/fimmu.2026.1785523

**Published:** 2026-04-14

**Authors:** Qin Yang, Yuanhui Song, James F. Markmann, Ji Lei

**Affiliations:** 1 Penn Transplant Institute, Department of Surgery, Hospital of the University of Pennsylvania, Philadelphia, PA, United States; 2 Center for Transplantation Sciences, Massachusetts General Hospital, Harvard Medical School, Boston, MA, United States

**Keywords:** immune rejection, immune engineering, immunomodulation, islet microenvironment, type 1 diabetes, β-cell replacement

## Abstract

Type 1 diabetes (T1D) is driven by autoimmune destruction of pancreatic β-cells and remains incurable despite major advances in insulin delivery and glucose-monitoring technologies. Transplantation of primary islets or stem cell-derived β-like cells offers a promising route to physiological glycemic control; however, durable engraftment remains limited by complex immune rejection. Unlike classical solid organ transplantation, β-cell replacement in T1D confronts a uniquely intertwined set of immunological barriers, including innate inflammatory activation, adaptive alloimmunity, persistent humoral responses, and recurrent autoimmune memory, further exacerbated by as-yet undefined factors that disrupt the native islet microenvironment. These overlapping effector pathways help explain why single-axis immunosuppressive or physical shielding strategies have not achieved long-term protection. In this review, we synthesize current mechanistic insights into the immune processes that limit β-cell graft survival and organize emerging therapeutic strategies according to the rejection pathways they target. We discuss advances in graft-intrinsic immune engineering, local graft-adjacent immunomodulation, and systemic immune interventions aimed at mitigating innate inflammation, cellular and humoral immunity, and autoimmune recurrence. We further highlight translational progress, safety considerations, and regulatory challenges associated with these approaches. Collectively, this mechanistic perspective provides a rational framework for designing coordinated immunomodulatory strategies to enable durable, immune-compatible β-cell replacement for T1D.

## 1 Introduction

Type 1 diabetes (T1D) is a chronic autoimmune disease characterized by immune-mediated destruction of insulin-producing pancreatic β-cells, resulting in lifelong dependence on exogenous insulin (1, 2). Although glucose-monitoring technologies and automated insulin delivery systems have significantly improved disease management, long-term glycemic control remains imperfect. Patients continue to face risks of severe hypoglycemia, progressive microvascular and macrovascular complications, and reduced life expectancy and quality of life (3–6). With the global prevalence of T1D projected to exceed 13 million individuals by 2040 (7, 8), there is an urgent need for disease-modifying and restorative therapeutic approaches.

Cell replacement strategies, including transplantation of primary human islets and stem cell-derived β-like cells, offer a promising route toward physiological glucose regulation and potential insulin independence (9–11). Clinical trials have shown that islet transplantation can restore endogenous insulin secretion and prevent severe hypoglycemia in appropriately selected individuals (12–14). However, despite this therapeutic promise, durable β-cell graft survival in T1D remains difficult to achieve, largely because immune rejection involves multiple overlapping mechanisms that extend beyond the reach of any single protective strategy (15, 16). Current clinical protocols depend heavily on systemic immunosuppression, which carries risks of infection, malignancy, and drug toxicity, while still failing to prevent gradual graft attrition in many recipients (17–22). Transplanted β-cells are simultaneously subjected to early innate inflammation, adaptive alloimmune T-cell responses, antibody-mediated humoral injury, and recurrent autoimmune memory, all of which are further intensified by disruption of the native islet microenvironment. These redundant and convergent effector pathways impose a level of biological complexity that cannot be adequately controlled by single-axis interventions, whether encapsulation devices, conventional immunosuppressive drugs, or isolated gene edits (2, 19, 20, 22).

Historically, efforts to improve graft outcomes have centered on pharmacological immunosuppression, aimed primarily at broad T-cell inhibition (23). Although effective against acute rejection, this approach is nonspecific and poorly suited for lifelong use in young patients with T1D (9, 24, 25). Increasing recognition of these limitations has driven a conceptual transition from systemic immunosuppression toward more targeted, multiscale immune reprogramming strategies. Rather than targeting a single dominant mechanism, emerging approaches seek to coordinate immune control across distinct spatial and functional levels (26–32). At the graft level, graft-intrinsic immune engineering aims to reduce antigen visibility and limit immune effector pathways. At the tissue level, local graft-adjacent immunomodulation focuses on reconstructing a tolerogenic microenvironment through regulatory immune cells, supportive stromal elements, and immuno-instructive biomaterials. At the organismal level, systemic immune reprogramming attempts to reshape pathological immune memory and modulate host immune tone. Together, these approaches reflect the multifaceted immune mechanisms that govern β-cell graft rejection in T1D.

In this Review, we synthesize the immune mechanisms that limit β-cell graft survival and organize contemporary therapeutic strategies according to the rejection pathways they are designed to target. By integrating advances in immune-evasive and tolerogenic gene engineering, co-transplantation-based immunomodulation, and systemic tolerance strategies with recent clinical progress in stem cell-derived β-cell replacement, we aim to outline key design principles, translational bottlenecks, and future directions necessary for developing durable, immune-compatible cell therapies for T1D.

## 2 Mechanisms of graft rejection in T1D

**Figure 1:**
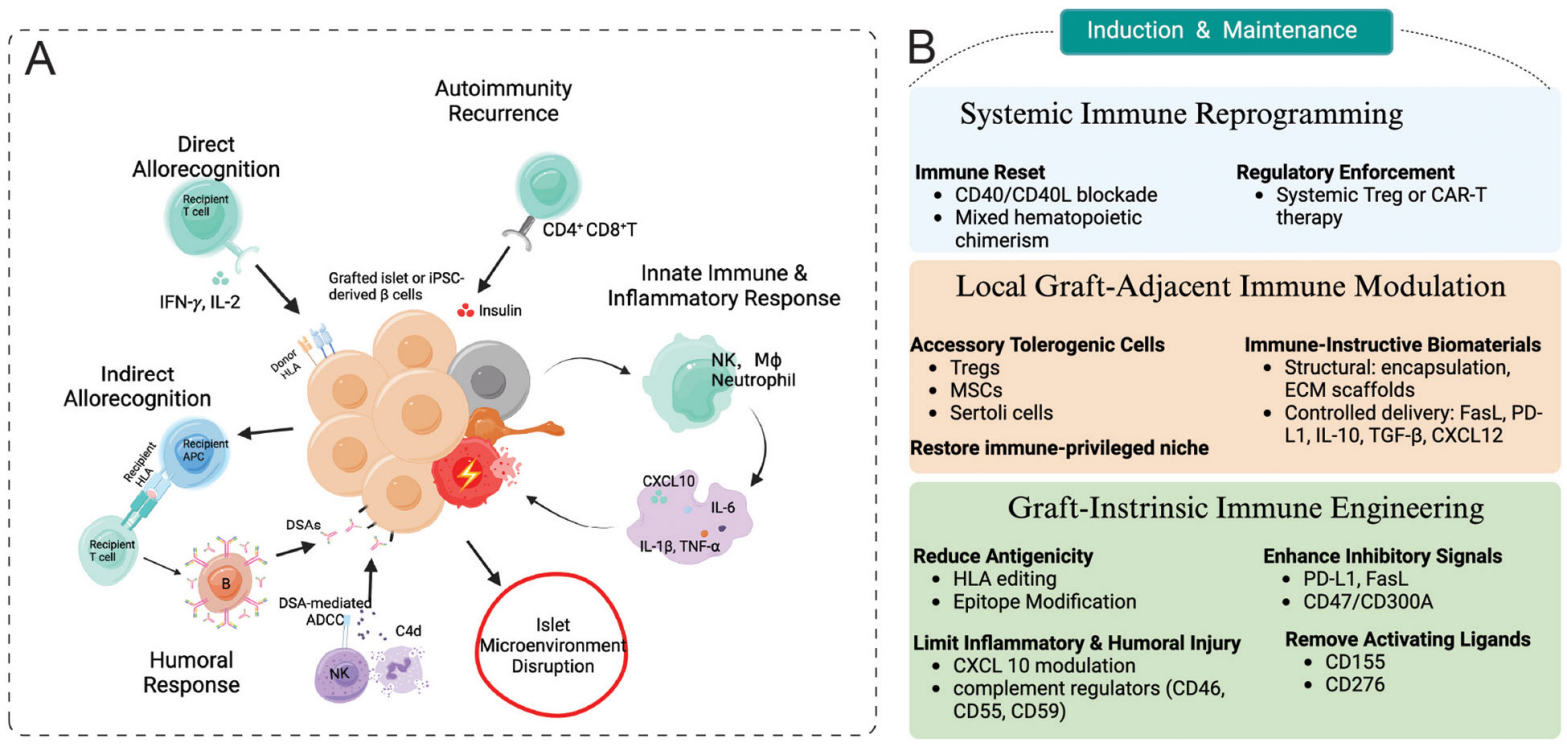
Immune barriers to β-cell replacement graft injury and layered strategies for immune modulation in type 1 diabetes. **(A)** Major immune mechanisms driving β-cell graft injury are illustrated, including direct and indirect allorecognition, recurrent autoimmunity, innate inflammatory activation, humoral immune injury, and disruption of the native islet microenvironment. These pathways collectively promote inflammatory injury and progressive graft dysfunction. **(B)** Layered immune modulation strategies designed to overcome these barriers. Graft-intrinsic immune engineering reduces antigenicity and susceptibility to innate and humoral attack, local graft-adjacent modulation shapes the immune microenvironment through accessory cells and biomaterials, and systemic immune reprogramming supports tolerance induction and long-term graft maintenance.

β-cell replacement in T1D encounters a uniquely complex immune response that extends beyond the challenges of classical allogeneic transplantation (21). Unlike solid-organ grafts, transplanted β-cells must withstand not only alloimmune recognition driven by human leukocyte antigen (HLA) mismatch (33, 34), but also pre-existing autoimmune memory, potent innate inflammatory activation at implantation, antibody-mediated humoral injury, and disruption of the native islet microenvironment. These mechanisms do not act in isolation but instead form a temporal and mutually reinforcing rejection cascade, in which early innate inflammatory injury initiates and amplifies subsequent adaptive alloimmune, autoimmune, and humoral responses (**Figure 1**). Understanding this multifaceted process is essential for the rational design of effective immunomodulatory strategies.

### 2.1 Innate immune and inflammatory responses

Innate immune activation is a dominant driver of early graft loss following β-cell transplantation and typically precedes the full engagement of the adaptive immune system (22, 35). Within minutes to hours of implantation, innate effector cells, including neutrophils, macrophages, and natural killer (NK) cells, are rapidly recruited to the graft site. These cells release pro-inflammatory mediators such as interleukin-1β (IL-1β), tumor necrosis factor-α (TNF-α), and interleukin-6 (IL-6), which impair β-cell function, disrupt insulin secretion, and promote apoptotic and necrotic cell death (36). This early innate response is particularly pronounced in the clinical standard of intraportal islet infusion, where transplanted islets come into immediate contact with blood, triggering the instant blood-mediated inflammatory reaction (IBMIR) (37). IBMIR involves activation of the coagulation and complement pathways, platelet aggregation, endothelial activation, and rapid leukocyte infiltration, leading to destruction of a substantial fraction (~50%) of the transplanted islet mass within hours (38, 39). Beyond reducing effective graft mass, IBMIR establishes a highly immunogenic environment that accelerates subsequent adaptive immune activation (19, 22).

Concurrently, hypoxia, ischemic injury, and mechanical stress induce β-cell injury during and after transplantation. Dying cells release danger-associated molecular patterns (DAMPs) that engage Toll-like receptors and other pattern-recognition receptors on macrophages, endothelial cells, and recruited innate immune cells. This sensing initiates a self-amplifying inflammatory loop that sustains cytokine production, enhances antigen presentation, and promotes lymphocyte trafficking into the graft (17, 18, 22). NK cells play a particularly important role during this early window. In the absence of sufficient inhibitory signaling, such as following downregulation of HLA class I, NK cells recognize stressed or “missing-self” β-cells and mediate cytotoxicity via perforin-granzyme pathways or, when antibodies are present, through antibody-dependent cellular cytotoxicity(ADCC) (48, 59–62). Macrophages further amplify graft injury through phagocytosis, nitric oxide production, and pro-inflammatory cytokine secretion, linking innate cytotoxicity to downstream T-cell priming (35, 63–65).

In this context, beyond apoptosis and necrosis associated with ischemic injury, metabolically driven non-apoptotic cell death pathways such as ferroptosis may further shape early graft vulnerability. Pancreatic β-cells possess limited antioxidant capacity and exhibit heightened sensitivity to disruptions in iron and redox homeostasis, rendering them susceptible to iron-dependent lipid peroxidation under hypoxic and inflammatory stress (66). Ferroptosis-like injury is intrinsically pro-inflammatory, promoting the release of oxidized lipid species and other DAMPs that engage innate immune sensing pathways (67). These signals may amplify early macrophage activation and NK-cell recruitment, reinforcing inflammatory feedback loops that contribute to initial graft attrition. Collectively, these innate inflammatory processes account for a substantial proportion of immediate graft attrition and form a critical platform for subsequent alloimmune, autoimmune, and humoral responses. Early innate injury lowers the activation threshold for effector T cells, increases the availability of antigen for indirect allorecognition, and heightens the vulnerability of β-cells to cellular and antibody-mediated attack. These features position innate immunity as a central therapeutic target for early graft protection through strategies such as localized immunomodulatory biomaterials, chemokine pathway blockade, complement inhibition, and NK cell-targeted immune engineering (57, 68).

### 2.2 Direct and indirect allorecognition

Mismatch between donor and recipient HLA molecules is a central driver of immune recognition of allogeneic primary islets as well as stem cell-derived β-like cells (33, 43). HLA genes, located within the major histocompatibility complex (MHC), encode highly polymorphic class I and class II molecules that present peptides to T cells (33, 34).

Class I molecules, including HLA-A, -B, and -C as well as the non-classical HLA-E, -F, and -G, are expressed on nearly all nucleated cells and present intracellularly derived peptides to CD8^+^ T cells. They consist of a 44-kDa α-chain associated with β2-microglobulin (34, 69–71).

Class II molecules , including HLA-DR, -DP, and -DQ, are primarily expressed on professional antigen-presenting cells (APCs) and present peptides derived largely from exogenous antigens to CD4^+^ T cells via an α/β heterodimer of approximately 32 and 28 kDa, respectively (33, 34, 69, 72).

Because of extensive HLA polymorphism, full donor-recipient matching is rare, and allograft transplantation frequently triggers robust T-cell activation (34, 73, 74).

Direct alloimmune responses remain a major driver of early graft loss. In this pathway, recipient CD8^+^ and CD4^+^ T cells recognize intact donor MHC class I and class II molecules, respectively, displayed on donor-derived APCs (75). This highly efficient mode of antigen recognition triggers rapid T-cell activation, perforin-granzyme-mediated cytotoxicity, and secretion of pro-inflammatory cytokines such as IL-2 and IFN- γ, contributing to acute graft rejection (76). In contrast, indirect allorecognition is mediated by recipient APCs, which internalize donor-derived proteins, process them into short peptides, and present donor alloantigen to naïve and memory CD4^+^T cells. Full CD4^+^ T-cell activation requires co-stimulatory signaling through CD80/CD28 and CD40/CD154, promoting the production of IL-2, IFN- γ, IL-4, IL-5, and IL-13 (19, 20). These signals support T-cell expansion and drive T cell-dependent B-cell differentiation into antibody-secreting plasma cells (34). The indirect pathway is particularly relevant to chronic rejection and the generation of donor-specific antibodies (DSA), which can accelerate long-term graft deterioration (18, 75).

### 2.3 Recurrent autoimmunity

Unlike most transplantation settings, β-cell replacement in T1D must contend with pre-existing autoimmunity memory directed against endogenous β-cell antigens (2, 77). Autoreactive CD4^+^ and CD8^+^ T cells specific for insulin, glutamic acid decarboxylase 65 (GAD65), insulinoma-associated antigen-2 (IA-2), and zinc transporter 8 (ZnT8) persist long after clinical diagnosis and can rapidly infiltrate transplanted grafts (2, 77–79). These memory T cells mediate antigen-specific cytotoxicity independently of alloantigen recognition, enabling autoimmune-mediated graft loss even in the absence of HLA mismatch (19, 21, 22, 79).

In parallel, B cells contribute to recurrent autoimmunity through the generation of circulating islet autoantibodies (80). Although autoantibodies are not the primary mediators of β-cell destruction, they amplify tissue injury through immune complex formation, complement activation, and Fc receptor-mediated effector pathways (60). Autoimmune graft loss has been observed in syngeneic transplantation models, demonstrating that suppression of alloimmunity alone is insufficient for durable graft protection in T1D (81–83).

Autoimmune memory is particularly resistant to conventional immunosuppression. Memory T cells exhibit reduced dependence on co-stimulatory signals, heightened effector function, and rapid tissue homing, rendering them far less susceptible to calcineurin inhibitors and corticosteroids (84). Moreover, inflammatory cues generated during early graft injury, such as IL-1β, IFN-γ, and TNF-α, can further lower activation thresholds for autoreactive lymphocytes, establishing a feed-forward loop between innate inflammation and adaptive autoimmunity (85).

Together, these features define recurrent autoimmunity as a non-redundant and persistent immune barrier distinct from classical alloimmune rejection. Consequently, effective β-cell replacement strategies in T1D must both suppress alloreactive immunity and actively restrain autoreactive memory. This requirement provides the mechanistic rationale for the development of immune checkpoint-based evasion strategies, regulatory T-cell therapies, and tolerogenic microenvironment engineering discussed in subsequent sections.

### 2.4 Humoral rejection and antibody-dependent cytotoxicity

Humoral immunity contributes to both acute and chronic graft injury in β-cell replacement, largely through the development of DSA (60). These antibodies arise predominantly via the indirect allorecognition pathway, in which CD4^+^ T cell-dependent B-cell activation drives class switching and plasma cell differentiation (86). DSAs bind donor HLA or other graft-expressed antigens and initiate effector pathways that mediate graft injury. A major component of antibody-mediated rejection is complemention activation, in which DSA binding recruits C1q and activates the classical pathway, leading to C4d deposition, membrane attack complex formation, and endothelial injury. Complement-mediated damage impairs islet engraftment and increases susceptibility to subsequent cellular immune attack (60).

Antibodies also engage innate effector cells through Fcγ receptor-dependent mechanisms. NK cells mediate ADCC via perforin-granzyme pathways, while macrophages phagocytose opsonized targets and release inflammatory mediators (18, 60). These pathways are especially injurious when stressed β-cells exhibit altered or insufficient HLA class I expression, reducing inhibitory signaling and increasing susceptibility to NK-cell recognition. Because DSA production can occur even under conventional immunosuppression, humoral immunity represents a distinct and non-redundant barrier to long-term graft function. Effective β-cell replacement strategies must therefore consider approaches that limit DSA formation, block complement activation, or attenuate Fc receptor-mediated injury (87, 88).

### 2.5 Disruption of the native islet microenvironment

In the native pancreas, islets reside within a highly specialized stromal and vascular microenvironment that provides partial protection from immune surveillance while supporting efficient oxygenation, nutrient delivery, and metabolic signaling (60, 89). Pericytes, extracellular matrix components, and fenestrated endothelial cells together establish a tightly regulated interface that regulates immune cell access and dampens inflammatory amplification (90, 91).

By contrast, transplant β-cells, whether delivered intraportally, subcutaneously, intramuscularly, or within macro- or microencapsulation devices, are displaced from this native microenvironment and become directly exposed to circulating immune cells, inflammatory cytokines, and metabolic stress (91, 92). Intraportal transplantation, although clinically established, exposes islets to steep oxygen gradients, shear stress, and continuous immune surveillance within the hepatic sinusoids (93, 94). Extrahepatic sites, including subcutaneous or intramuscular locations, often suffer from delayed revascularization and hypoxia, further sensitizing grafts to immune-mediated injury (80).

Disruption of the native islet microenvironment amplifies the impact of innate inflammation, adaptive cellular immunity, and humoral rejection, effectively lowering the threshold for graft damage across all pathways described in Sections 2.1-2.4. This vulnerability also helps explain why systemically effective immunosuppression or graft-intrinsic immune evasion alone is often insufficient for long-term graft durability. These limitations have motivated the development of biomaterial-based and niche-modulation strategies designed to recreate immune-instructive microenvironments around transplanted β-cells. By physically separating grafts from circulating immune effectors, locally delivering immunomodulatory cues, and promoting rapid vascular integration, engineered niches aim to restore key elements of immune privilege that are otherwise lost during transplantation (46, 80, 95–97). This concept provides an essential mechanistic foundation for biomaterial-based, and niche-modulation strategies for immune protection discussed in later sections.

## 3 Graft-intrinsic immune engineering

Graft-intrinsic immune engineering aims to reduce the immunogenicity of transplanted β-cells while preserving endocrine identity and glucose-responsive insulin secretion (98). Advances in CRISPR-Cas-based multiplex genome editing now enable coordinated manipulation of antigen presentation pathways, immune checkpoint pathways, chemokine and cytokine responsiveness, and antibody-interacting cell surfaces molecules. As a result, the field has shifted from single-gene edits toward combinatorial immune evasion strategies designed to simultaneously blunt T-cell-, natural killer (NK) cell-, and antibody-mediated rejection (99).

A central design challenge is that reducing immune visibility to T cells, particularly through HLA modulation, can increase susceptibility to innate immune attack via the “missing-self” mechanism (100–103). Effective immune engineering therefore requires balanced, bidirectional control of adaptive and innate immunity rather than simple antigen deletion. This section summarizes the major genetic strategies currently used to generate immune-evasive β-cell grafts.

### 3.1 HLA engineering to evade T-cell and NK-cell recognition

A primary strategy for attenuating T-cell-mediated rejection is the disruption of HLA class I and class II expression to prevent direct recognition by CD8^+^ and CD4^+^ T cells, respectively (81, 104). Deletion of β2-microglobulin (B2M) eliminates surface expression of classical HLA class I molecules and effectively blocks CD8^+^ T-cell recognition (105–108). However, complete loss of HLA class I also removes critical inhibitory ligands for NK cells, rendering engineered cells highly susceptible to missing-self-mediated NK cytotoxicity (109, 110). To restore NK inhibition while maintaining reduced T-cell visibility, several complementary strategies have been developed:

1. Enforced expression of non-classical HLA molecules.

Overexpression of HLA-E and/or HLA-G provides inhibitory ligands for NK receptors such as NKG2A, KIR2DL4, and LILRB1, suppressing NK activation without restoring classical T-cell recognition (41, 42, 109, 111).

2. Selective deletion of polymorphic class I alleles.

Editing strategies that delete HLA-A and HLA-B while retaining HLA-C reduce allogeneic CD8^+^ T-cell recognition while maintaining partial NK-cell inhibition through inhibitory KIR-HLA-C interactions. This configuration generates a “pseudo-self” inhibitory profile that mitigates both T-cell-mediated rejection and NK-cell activation (43). However, because NK inhibition depends on KIR-HLA matching and varies across individuals, some hypoimmune engineering strategies instead retain or introduce HLA-E, which engages the broadly expressed inhibitory receptor NKG2A to provide more consistent NK-cell inhibition (111).

3. Disruption of HLA class II expression.

CD4^+^ T-cell activation can be attenuated by eliminating HLA class II expression through disruption of transcriptional regulators such as CIITA or RFX5, thereby limiting indirect allorecognition-driven CD4^+^ T-cell activation (44, 47, 71, 105, 112).

4. Combined HLA-I and HLA-II editing.

Multiplex genome editing targeting both class I and class II pathways can suppress both arms of adaptive cellular immunity (25, 44, 47, 74, 78, 113). Importantly, such edits have been successfully introduced into human pluripotent stem cells and maintained through differentiation into insulin-producing β-like cells, with preservation of glucose-stimulated insulin secretion and endocrine identity (41, 47, 114–117). These results establish HLA engineering as a viable foundation for universal, donor-independent β-cell products.

Despite these advances, precise tuning of NK-T-cell balance remains a major constraint. Insufficient NK inhibition risks immediate innate-mediated graft destruction, whereas excessive inhibition may impair antitumor surveillance or promote viral susceptibility in long-term graft recipients (41, 44, 48, 78, 87, 114, 117–119). Beyond non-classical HLA restoration and CD47-mediated innate cloaking, emerging strategies such as engagement of inhibitory receptors, including CD300A, further expand the repertoire of NK-regulatory approaches (120). Together, these complementary yet incomplete solutions underscore that no single modification can fully balance immune evasion with safety. Consequently, combinatorial and context-dependent design principles are likely required, as discussed in later sections.

### 3.2 Expression of immune checkpoint ligands and self-recognition cues

Engineering β-cells to express inhibitory ligands provides a complementary strategy for suppressing alloimmune, autoimmune, and innate cytotoxic responses without relying solely on HLA deletion. Checkpoint molecules and “self-recognition” cues increase activation thresholds across T cells, NK cells, and myeloid cells, thereby attenuating inflammatory amplification at the graft site (121–123).

Checkpoint inhibition of T cells. Overexpression of PD-L1 on β-like cells suppresses CD4^+^ and CD8^+^ T-cell proliferation, cytokine release, and cytotoxicity by engaging PD-1 (45, 124–128). Co-expression of CTLA4-Ig, which blocks CD80/CD86-CD28 co-stimulation, further dampens early T-cell priming and late effector responses (86, 129–131). In contrast to activation-threshold modulation, localized presentation of Fas ligand (FasL/CD95L), through islet surface engineering or co-transplantation platforms, has been explored to induce apoptosis of infiltrating activated T cells and promote graft-localized immune tolerance (54, 132, 133).

Innate self-recognition signals. Expression of CD47 engages SIRPα on macrophages to inhibit phagocytosis and reduce myeloid-driven inflammation. Non-classical HLA molecules such as HLA-E and HLA-G function as checkpoint-like self-recognition ligands by engaging NKG2A and LILRB1/2, restraining NK-cell activation and promoting tolerogenic myeloid phenotypes (38, 44, 134–136).

Combinatorial engineering. Because individual ligands confer only partial protection, several platforms now combine PD-L1, CD47, and non-classical HLA molecules to concurrently inhibit T-cell cytotoxicity, NK-cell activation, and myeloid inflammation (137). Checkpoint-enhanced β-like cells generally retain endocrine identity and glucose responsiveness, though long-term safety, particularly with respect to antitumor surveillance, remains an important consideration.

### 3.3 Removal of activating ligands and inflammatory amplifiers

In addition to suppressing antigen presentation and enhancing inhibitory signaling, immune evasion can be reinforced by removing activating ligands that directly stimulate cytotoxic immune receptors. Stem cell-derived β-cells express multiple stress-induced molecules capable of engaging activating receptors on NK cells and cytotoxic T lymphocytes. These pathways can override inhibitory cues and mediate graft destruction even when HLA expression is reduced or checkpoint ligands are present. Genome-scale CRISPR screening has identified CD155 (PVR) and CD276 (B7-H3) as dominant NK-activating ligands that signal through receptors such as DNAM-1, promoting NK-cell cytotoxicity (48). Targeted deletion of these ligands markedly reduces NK activation and enhances survival of engineered β-cell grafts. Because NK receptor repertoires vary among individuals, optimal protection often requires multiplex disruption of activating ligands alongside restoration of inhibitory signals such as HLA-E or CD47 (49).

Beyond direct NK-activating cues, chemokine-driven immune recruitment represents an additional genetically tractable vulnerability. Deletion of CXCL10, a key chemoattractant for CXCR3^+^ effector T cells, limits early lymphocyte infiltration and dampens inflammatory amplification around the graft (49).

Together, removal of activating ligands and modulation of chemokine signals highlight that effective immune evasion requires controlling both immune detection and immune access. From a translational standpoint, ligand and chemokine editing allows fine-tuning of immune sensitivity without enforcing complete invisibility, potentially preserving host defense while reducing acute cytotoxic risk.

### 3.4 Shielding from humoral and complement-mediated injury

Humoral rejection remains a major vulnerability for β-cell grafts, particularly in allogeneic or previously sensitized recipients. Even when T-cell and NK-cell pathways are suppressed, DSAs can bind surface alloantigens and trigger complement activation, ADCC, and Fc receptor-mediated inflammation (29, 59). To mitigate these pathways, engineered β-cells have been modified to express complement regulatory proteins such as CD46, CD55, and CD59, which inhibit C3 and C5 convertase formation and block membrane attack complex assembly (50). These edits reduce complement-mediated lysis and dampen inflammatory bystander damage. Additional strategies include surface engineering approaches that limit IgG binding or prevent C1q recruitment, thereby reducing activation of the classical complement pathway and subsequent ADCC.

Because complement activation amplifies NK-cell and macrophage responses, humoral shielding enhances the efficacy of HLA editing, checkpoint ligand expression, and innate inhibitory cues. Importantly, complement regulation does not require complete antigen invisibility and can be combined with other edits without impairing β-cell differentiation or function. Collectively, humoral and complement shielding provides a modular and scalable mean to reduce antibody-mediated injury and increase graft resilience in sensitized or alloimmune environments.

### 3.5 Autoimmune epitope modification and control of β-cell autoantigenicity

Unlike most allogeneic grafts, β-cell replacement in T1D must overcome pre-existing autoimmune memory directed against insulin, GAD65, IA-2, and ZnT8 (2, 77, 80, 138). These autoreactive CD4^+^ and CD8^+^ T cells can target transplanted β-cells regardless of their source or HLA compatibility. Accordingly, for stem cell-derived β-cells, immune engineering must attenuate autoimmune recognition without compromising β-cell maturation, identity, or glucose responsiveness (86).

Selective deletion or modification of dominant autoimmune epitopes has been explored to reduce T-cell targeting while maintaining essential endocrine functions. Precise alteration of the immunodominant insulin B-chain epitope (InsB9-23), including modification of critical T-cell receptor contact residues, has been shown to modulate diabetogenic T-cell recognition in NOD models while preserving functional insulin expression and activity. These studies provide proof-of-principle that epitope-focused deimmunization can decouple autoimmune recognition from β-cell function (51). Alternatively, immune recognition can be reduced by dampening inflammatory stress-response pathways that amplify antigen presentation. Interferon- γ-driven signaling, particularly through STAT1 activation, induces HLA upregulation and promotes β-cell susceptibility to autoimmune attack. Strategies that attenuate IFN- γ responsiveness or limit inflammation-induced HLA induction have been shown to reduce immune targeting while preserving basal antigen presentation and cellular homeostasis necessary for β-cell survival (52, 139, 140).

A central challenge in these approaches is avoiding dedifferentiation or metabolic impairment. Excessive antigen removal or broad suppression of stress-response pathways may disrupt insulin processing, transcriptional identity, or adaptive stress resilience. For this reason, autoimmune-directed modifications are typically integrated with complementary strategies, such as checkpoint ligand expression, HLA modulation, or local tolerogenic microenvironments, to provide coordinated immune protection while maintaining β-cell identity and function. Effective shielding from recurrent autoimmunity therefore requires a calibrated balance between immune evasion and preservation of essential β-cell biology.

Compared with primary islets, stem cell-derived β-cells exhibit distinct HLA and immune checkpoint regulatory dynamics, including differential PD-L1 expression under cytokine exposure (117). More broadly, evidence from pluripotent stem cell-derived systems indicates that incomplete maturation can shift the balance between inhibitory HLA class I signaling and activating NK ligands, increasing susceptibility to NK-cell-mediated cytotoxicity (141). In parallel, metabolic immaturity, including incomplete mitochondrial and redox maturation, may further predispose grafts to functional instability under inflammatory stress. These observations underscore that maturation state and graft-intrinsic immune engineering are mechanistically intertwined determinants of durable graft survival.

### 3.6 Combinatorial immune-evasive design principles

Experience from multiplex CRISPR engineering, immune-privileged grafts, and early clinical translation indicates that no single modification can fully prevent rejection. Instead, durable immune evasion depends on coordinated, multi-axis editing that simultaneously regulates antigen visibility, inhibitory signaling, innate activation thresholds, and susceptibility to humoral injury. A key design principle is maintaining a dynamic balance between immune pathways:1) Reducing HLA expression protects against T-cell recognition but increases susceptibility to NK-cell activation. 2) Introducing inhibitory ligands (e.g., HLA-E, CD47, PD-L1) suppresses NK or T-cell responses; however, excessive inhibition may compromise host antitumor or viral surveillance. 3) Eliminating activating ligands (e.g., CD155 or NKG2D ligands) can reduce NK cytotoxicity but must be carefully integrated with strategies for complement shielding and chemokine modulation. Thus, combinatorial immune-evasion engineering is not additive but interactive. Each edit shifts immune activation thresholds, and optimal designs therefore integrate multiple layers of controls, including reduced antigenicity (HLA editing, epitope modification), enhanced inhibitory cues (PD-L1, CD47, HLA-E/G), elimination of activating ligands (CD155, CD276), suppression of inflammatory recruitment (e.g., CXCL10 or cytokine-response modulation), and protection from humoral attack (CD46, CD55, CD59).

As immune evasion becomes increasingly effective, safety emerges as an intrinsic design constraint rather than a secondary consideration. Extensive suppression of immune recognition through HLA deletion, checkpoint ligand expression, or removal of activating signals can impair tumor surveillance and antiviral defense, necessitating built-in mechanisms for graft control. To address this trade-off, several platforms have incorporated inducible safety switches, such as inducible caspase-9 (iCasp9), enabling rapid and selective elimination of graft cells in the event of uncontrolled proliferation, transformation, or infection (57). From a design perspective, these safeguards allow immune evasion to be tuned rather than maximized, reinforcing the principle that combinatorial immune-evasive engineering must balance immune protection with reversibility, control, and long-term safety.

In summary, combinatorial immune-evasive engineering reframes graft protection as a systems-level design problem in which innate, adaptive, and humoral pathways must be balanced rather than eliminated. This principle underlies current efforts to develop immune-compatible β-cell therapies.

## 4 Local graft-adjacent immunomodulation

While gene engineering strategies modify graft-intrinsic immunogenicity, co-transplantation–based immunomodulation seeks to actively reshape the local immune environment surrounding the graft (46, 128, 129). This approach leverages immunoregulatory cells delivered alongside β-cells grafts to suppress effector immunity, reinforce tolerance, and promote a protective stromal and cytokine milieu. In contrast to systemic immunosuppression, co-transplantation strategies aim to achieve localized, graft-restricted immune regulation with reduced off-target toxicity. Co-transplanted immunoregulatory cells can modulate multiple components of the rejection cascade outlined in Section 2, including dampening innate inflammation (Section 2.1), suppressing alloreactive and autoreactive T cells (Sections 2.2 and 2.3), and indirectly limiting humoral immune activation (Section 2.4). These effects are mediated through direct cell-cell interactions, secretion of tolerogenic cytokines, metabolic regulation of immune responses, and remodeling of the stromal microenvironment (54, 55, 128, 132, 142, 143). As such, co-transplantation provides a dynamic and adaptive model of immune control that is highly complementary to static gene-engineering approaches.

### 4.1 Regulatory T cells, mesenchymal stromal cells, and other tolerogenic accessory cells

#### 4.1.1 Regulatory T cells (Tregs)

Tregs suppress effector T-cell activation, modulate macrophage and dendritic-cell phenotypes, and promote local tolerance through IL-10, TGF-β, and cell-contact-dependent mechanisms (56, 144). Engineered CAR-Tregs can selectively localize to grafts by recognizing alloantigen or β-cell-associated targets. Preclinical studies demonstrate that CAR-Tregs prolong islet graft survival and outperform polyclonal Tregs by providing targeted and durable suppression (57, 145). New designs targeting broadly expressed ligands such as OX40L further enhance their function in inflamed graft microenvironments (146). Key translational challenges include achieving stable *in vivo* persistence, maintaining phenotype in pro-inflammatory contexts, and overcoming the negative effects of standard immunosuppressive drugs on Treg survival.

#### 4.1.2 Mesenchymal stromal cells

MSCs exert broad immunoregulatory effects through secretion of IL-10, TGF-β, PGE2, IDO, and HGF, suppressing T-cell proliferation, inhibiting dendritic-cell maturation, polarizing macrophages toward M2 phenotypes, and attenuating NK-cell cytotoxicity (147–151). In islet transplantation models, MSC co-transplantation reduces IBMIR-associated early graft loss, enhances revascularization, and remodels extracellular matrix architecture (150, 152). MSC-derived trophic and angiogenic factors also improve β-cell metabolic fitness and resistance to inflammation (119, 130, 153). Limitations include donor variability, short persistence, and context-dependent immunomodulation, driving interest in pre-activated, genetically engineered, or extracellular-vesicle-based MSC therapies (154).

#### 4.1.3 Other tolerogenic accessory cells

Additional candidates include tolerogenic dendritic cells (tolDCs), myeloid-derived suppressor cells (MDSCs), type 1 regulatory T cells (Tr1 cells), and Sertoli cells, each capable of suppressing effector immunity and promoting regulatory phenotypes via cytokine production, inhibitory ligand expression, or metabolic immune regulation (155, 156). Although at earlier stages of development, these cell types expand the repertoire of graft-adjacent strategies capable of modulating multiple arms of the rejection cascade.

Together, these immunoregulatory cell platforms form a flexible toolkit for constructing localized tolerogenic niches that complement and synergize with graft-intrinsic immune engineering.

### 4.2 Biomaterial, encapsulation, and niche modulation

Biomaterials and encapsulation devices offer a structural means to shape the immune microenvironment. Hydrogels, alginate-based microcapsules, and macro-encapsulation devices can physically separate grafts from circulating immune cells while permitting diffusion of nutrients, oxygen, and insulin (95, 96). Engineered biomaterials can also deliver local immunomodulatory cues, including controlled release of FasL, PD-L1, IL-10, TGF-β, CXCL12, or IDO-inducing factors, thereby dampening inflammation and promoting regulatory cell recruitment (157–159).

Niche modulation platforms aim to recreate elements of the native islet microenvironment, supporting rapid revascularization, reducing hypoxic stress, and attenuating DAMP-driven innate activation. Immune-instructive scaffolds incorporating extracellular matrix components or stromal cells provide additional regulation of macrophage and dendritic-cell behavior. While encapsulation reduces direct immune contact, trade-offs exist, including restricted oxygen diffusion may impair β-cell function, and long-term fibrosis can compromise device performance. Consequently, modern designs increasingly integrate ultra-thin barriers, vascularizing biomaterials, or degradable components to balance protection and physiological responsiveness.

Beyond serving as passive immune isolating barriers, next-generation biomaterials increasingly incorporate structural and topographical features that actively instruct local immune responses. Parameters such as pore architecture, surface chemistry, and mechanical stiffness can influence macrophage polarization, foreign body responses, and fibrotic remodeling, thereby shaping the inflammatory tone at the graft interface. Clinically advancing macroencapsulation systems in stem cell-derived β-cell transplantation, including retrievable and oxygenation-supported devices, reflect this shift by integrating vascular access, metabolic support, and controlled immune exposure within a unified architecture (160, 161). Improved oxygen delivery may further mitigate hypoxia-induced metabolic stress and DAMP release, attenuating early innate inflammatory activation. Recent replenishable revascularized platforms extend this concept by coupling immune compatibility with modular graft replacement strategies (162), positioning biomaterial design as an active component of immunomodulation rather than a purely physical barrier.

### 4.3 Integration and limitations of local niche-based immune modulation

Local niche-modulation strategies complement graft-intrinsic immune engineering by shaping the immediate immune microenvironment surrounding transplanted β-cells. Biomaterial platforms, immune-instructive scaffolds, and co-transplantation of immunoregulatory cells reduce early inflammation, limit effector cell access, and reinforce tolerogenic signaling at the graft interface, thereby increasing graft resilience under inflammatory or alloimmune stress (55).

However, because these effects are spatially restricted, niche-based strategies remain vulnerable to systemic immune activation, foreign-body responses, fibrosis, and impaired nutrient diffusion, limiting their durability as standalone interventions, particularly in sensitized or autoimmune settings. Local immune modulation functions as a dynamic buffering layer that dampens inflammatory amplification and stabilizes graft-host interactions but does not replace the need to control systemic immune memory or intrinsic graft immunogenicity. These approaches function as adaptive buffers that dampen inflammatory amplification without altering graft antigenicity.

## 5 Systemic immune reprogramming and immune reset strategies

In addition to graft-intrinsic engineering (Section 3) and local microenvironmental modulation (Section 4), systemic immune reprogramming aims to reshape the host immune system itself to induce durable, organism-level tolerance. These approaches seek to suppress autoreactive immune memory and prevent the generation of pathogenic alloreactive responses, thereby reducing long-term dependence on local graft protection. While potentially transformative, systemic immune reset carries greater biological risk and regulatory complexity, requiring careful calibration between tolerance induction, immune competence, and safety.

### 5.1 Systemic regulatory T-cell therapy

Adoptive transfer of ex vivo–expanded regulatory T cells (Tregs) is the most clinically advanced systemic tolerance strategy evaluated in autoimmunity and transplantation (163, 164). Unlike co-transplanted Tregs that act locally at the graft site, systemically infused Tregs circulate broadly, suppressing effector T-cell activation, modulating antigen-presenting cell function, and reinforcing peripheral tolerance across multiple immune interfaces (165). Both polyclonal and antigen-enriched Treg products have shown biological activity in early-phase trials (163, 164, 166). Antigen-specific approaches, targeting alloantigen or β-cell-associated epitopes, offer improved potency and tissue selectivity but face additional challenges in manufacturing consistency and phenotypic stability. Across studies, the major limitations include: limited *in vivo* persistence, susceptibility to inflammatory destabilization, and potential antagonism by standard immunosuppressive drugs that blunt Treg function (144). Thus, systemic Treg therapy is best viewed as an induction-phase tolerance adjunct, rather than a standalone alternative, complementing graft-intrinsic immune evasion and local immunoregulatory strategies. Complementary approaches aim to induce donor-specific Tregs *in vivo* by delivering nonimmunogenic donor antigen. Preclinical studies in nonhuman primates demonstrate that tolerogenic antigen presentation can expand regulatory T-cell populations *in vivo* and promote systemic immune regulation without extensive ex vivo manipulation (167).

In parallel, blockade of the CD40-CD40L co-stimulatory pathway has emerged as a complementary systemic immunomodulatory approach to limit early alloimmune priming. CD40 signaling licenses antigen-presenting cells and supports T-cell-dependent B-cell activation, linking cellular rejection with donor-specific antibody formation. Preclinical and translational studies (168, 169), including nonhuman primate kidney and islet transplantation models, demonstrate that CD40 or CD154 inhibition prolongs graft survival and attenuates humoral sensitization. Such strategies may be particularly suited to induction-phase immune control, facilitating early graft acceptance while reducing reliance on chronic systemic immunosuppression.

### 5.2 CAR-Tregs and precision systemic immune redirection

Chimeric antigen receptor-engineered Tregs (CAR-Tregs) extend the concept of adoptive Treg therapy by enabling antigen-directed suppression. CAR-Tregs can be engineered to recognize alloantigen, β-cell-associated antigens and inflammation-associated ligands, such as OX40L (146). Through antigen-specific homing and activation, CAR-Tregs limit effector T-cell responses and modify local antigen-presenting cells with greater precision than polyclonal Tregs (146, 170). Their improved tissue retention and resistance to bystander dilution make them attractive for autoimmune-prone settings such as T1D.

However, systemic deployment of CAR-Tregs introduces unique risks, including off-target immune suppression, prolonged persistence beyond the therapeutic window, phenotypic instability under inflammatory stress, and significant manufacturing and regulatory challenges. Accordingly, CAR-Tregs represent a promising tool for precision systemic immune redirection, but they are likely to function most effectively alongside graft-intrinsic and local tolerance strategies rather than as an isolated therapy.

### 5.3 Hematopoietic stem cell transplantation and mixed chimerism

Hematopoietic stem cell transplantation (HSCT) offers the most profound form of systemic immune reset, capable of re-establishing central tolerance by reshaping or replacing the host immune system. In transplantation, mixed hematopoietic chimerism, in which donor and recipient hematopoietic systems coexist, has emerged as a mechanism for inducing donor-specific tolerance through central deletion or functional silencing of alloreactive T-cell clones (171–173). In principle, HSCT and chimerism could eliminate autoreactive and alloreactive immune repertoires that threaten β-cell grafts. However, the clinical application of HSCT for T1D or β-cell replacement is constrained by major risks, conditioning-related toxicity, infection, graft-versus-host disease, impaired vaccine-derived immunity, and long-term malignancy risk.

Therefore, HSCT-based tolerance strategies currently serve primarily as proof-of-concept models that define the upper limit of what systemic immune reset can achieve. Their greatest value lies in informing the design of safer, non-myeloablative or targeted immune reset platforms rather than providing a scalable clinical solution (58, 174, 175).

### 5.4 Limitations, risks, and clinical boundaries of systemic immune reset

Despite their conceptual appeal, systemic immune reprogramming strategies are limited by fundamental biological, safety, and translational constraints (27, 28, 31). Biological risks include opportunistic infection, impaired tumor immunosurveillance, erosion of vaccine-derived immunity, and long-term immune dysregulation (176). These concerns are particularly relevant for durable or irreversible interventions such as CAR-Tregs or HSCT-based approaches.

The durability of tolerance also remains unresolved. Autoimmunity relapse, incomplete deletion of memory effector clones, and phenotypic instability of infused regulatory cells all undermine long-term outcomes. In addition, product heterogeneity, GMP manufacturing demands, high cost, and individualized dosing restrict widespread deployment. For these reasons, systemic immune reprogramming is unlikely to serve as a universal or isolated solution for β-cell replacement. Its most realistic clinical role lies in highly sensitized recipients, severe autoimmune recurrence, or as a temporary induction strategy that facilitates early graft acceptance before transitioning to graft-intrinsic and local protection.

Collectively, these limitations underscore why durable β-cell graft survival will require coordinated immunomodulatory strategies that address multiple, non-redundant rejection mechanisms. Effective approaches are likely to integrate graft-intrinsic immune engineering (Section 3), local microenvironmental immunomodulation (Section 4), and carefully bounded systemic immune reprogramming (Section 5), with the relative contribution of each tailored to recipient immune risk and clinical context. Accordingly, systemic immune reprogramming is best viewed as a bounded, context-dependent adjunct that complements graft-intrinsic and local immunomodulation rather than replacing them.

## 6 Clinical progress and translational pathways for β-cell replacement therapies

The immunomodulatory strategies discussed above (summarized in **Table 1**) are increasingly being evaluated in clinical b-cell replacement platforms using both primary islets and stem cell-derived b-like cells (28, 165, 177). To date, clinical efforts have explored a spectrum of translational approaches that differ in how immune protection is achieved, balancing graft function, safety, and durability.

**Table 1:** Immune-modulatory strategies for β-cell replacement.

Strategy category	Targeted immune mechanism (s)	Representative approaches	Key advantages	Key limitations/risks	Translational status
HLA editing	Direct and indirect allorecognition; NK-cell missing-self recognition	• HLA-I (B2M) knockout alone (40) • HLA-I knockout + HLA-E/G (41, 42) • Selective HLA-A/B deletion (43) • Combined HLA-I + HLA-II knockout (44)	Strong T-cell evasion	NK activation, viral risk	Preclinical
Checkpoint ligand expression	T-cell, NK-cell, and myeloid activation	• PD-L1 (45) • CTLA4-Ig (46) • CD47 (47) • HLA-E/G	Broad immune inhibition	Incomplete protection, safety risk	Preclinical
Activating ligand removal	NK-cell-mediated cytotoxicity	• CD155 (PVR) and CD276 (B7-H3) deletion (48) • CXCL10 deletion (49)	NK-specific control	Donor variability, partial protection	Preclinical
Humoral shielding	Humoral rejection	CD46, CD55, and CD59 overexpression (50)	Antibody resistance	Incomplete protection	Preclinical
Autoimmune epitope editing	Autoimmune recurrence	• InsB9–23 epitope modification (51) • IFN-γ-induced antigen presentation control (52)	Autoimmune-specific protection	Risk to β-cell identity, incomplete coverage	Preclinical
Local immune modulation	Local innate and adaptive immune activation	• Regulatory cell co-transplantation (53) • Immunomodulatory scaffolds (54) • Immune-instructive biomaterials and niches (55)	localized immune suppression	Spatial restriction, limited durability	Preclinical-early clinical
systemic immune reprogramming	Immune memory and tolerance regulation	• Polyclonal Treg therapy (56) • CAR-Tregs (57) • HSCT (58)	Potential for durable immune tolerance	safety risk, clinical complexity	Early clinical-experimental

Encapsulation-based platforms use macro- or microdevices to physically separate transplanted cells from host immunity while permitting diffusion of nutrients and insulin. These approaches prioritize safety and graft retrievability but continue to face persistent challenges, including diffusion limitations, hypoxia, foreign-body fibrosis, and incomplete immune isolation (95–97, 178). In parallel, unencapsulated stem cell-derived grafts supported by systemic or local immunosuppression have demonstrated robust engraftment and meaningful glycemic improvement in selected recipients. However, their broader application remains constrained by drug-related toxicity, infection risk, and long-term immunologic complications associated with chronic immunosuppression (179–181). More recently, immune-evasive or immunomodulated grafts have emerged as a rapidly advancing but still largely preclinical, translational direction. These approaches integrate graft-intrinsic immune engineering with local immunomodulation to reduce or potentially eliminate the need for lifelong systemic immunosuppression (9, 28, 47).

Across these approaches, early human studies have demonstrated measurable C-peptide production, improved glycemic stability, and partial insulin independence in select recipients, confirming the functional competence of stem cell-derived β-cells. However, durability remains variable, with immune-mediated attrition, device-related hypoxia, and fibrotic responses limiting long-term efficacy. Translational progress is further constrained by regulatory and manufacturing challenges, including product heterogeneity, lot-to-lot consistency, tumorigenicity risk, genomic stability after multiplex gene editing, and the absence of standardized potency assays. Patient selection criteria, endpoint definitions, and long-term surveillance strategies also continue to evolve.

Looking ahead, sustained clinical success is likely to depend on convergence rather than competition among these translational directions. Optimizing β-cell differentiation, implementing rational immune-evasive engineering, designing immune-supportive niches, and selectively integrating bounded systemic immunomodulation will need to proceed in a coordinated manner. Together, these considerations underscore the intrinsic biological and translational complexity of achieving durable β-cell graft function and immune compatibility in T1D.

## 7 Summary

Efforts to achieve durable β-cell graft survival in T1D have consistently demonstrated that targeting any single immune pathway is insufficient. Strategies focused solely on antigen deletion, such as removal of HLA class I to evade CD8^+^ T-cell recognition, frequently trigger compensatory immune responses, including NK-cell-mediated “missing-self” cytotoxicity (41, 111). Conversely, checkpoint ligand expression alone cannot fully prevent cytokine-driven antigen upregulation or antibody-mediated injury. These compensatory responses reflect the immune system’s intrinsic redundancy and underscore why durable graft survival requires coordinated immunomodulation rather than isolated interventions.

A defining challenge unique to T1D is the coexistence of alloimmune rejection and recurrent autoimmunity. Unlike most transplantation settings, recipients with T1D harbor long-lived autoreactive memory T cells directed against β-cell antigens such as insulin, GAD65, IA-2, and ZnT8 (84). These populations may persist independently of ongoing antigen exposure and can be relatively resistant to conventional tolerance mechanisms, creating a barrier distinct from classical allograft rejection. Experimental evidence from autoimmune models indicates that durable tolerance requires active reshaping of the host immune repertoire; for example, mixed hematopoietic chimerism with donor MHC class II expression can tolerize pre-existing autoreactive T cells through mechanisms that cannot be replicated by antigen invisibility alone (182, 183). Accordingly, strategies that address immune memory, rather than graft recognition alone, are likely to be necessary in autoimmune-prone recipients. Consistent with the need to reshape host immune memory, Tregs represent a clinically tractable strategy for systemic immune recalibration. Tregs possess functional attributes spanning multiple immune regulatory axes (184), including suppressive cytokine production, modulation of antigen-presenting cell function, and metabolic competition through high-affinity interleukin-2 consumption. This functional breadth has important implications for combinatorial design. Interventions targeting overlapping adaptive regulatory pathways may offer limited incremental benefit when layered onto effective Treg-mediated control, whereas strategies addressing distinct vulnerabilities, such as innate inflammatory injury, NK-cell recognition, or antibody-mediated damage, are more likely to provide complementary protection. Combinations that modify shared signaling pathways may also produce context-dependent outcomes influenced by localization, dosing, and immune activation state. Rational combinatorial design therefore requires consideration of mechanistic overlap, immune compartment specificity, and temporal deployment rather than simple accumulation of mechanistically overlapping interventions.

A critical distinction in combinatorial immunomodulation lies in the temporal separation between induction-phase immune control and long-term graft maintenance. Early after transplantation, inflammatory activation and antigen presentation create a narrow window during which alloimmune and autoimmune sensitization may become established. Induction strategies may therefore prioritize systemic interventions, such as regulatory T-cell therapy or co-stimulatory pathway blockade, to reset host immune responses and prevent early priming. However, sustained systemic immune manipulation is unlikely to be feasible for lifelong therapy. Durable graft protection will more likely depend on graft-intrinsic immune engineering and local niche modulation capable of maintaining immune compatibility without continuous immunosuppression.

As immune-evasive β-cell engineering becomes increasingly sophisticated, safety considerations inevitably emerge. Cells engineered to evade T-cell, NK-cell, and antibody-mediated recognition may also escape immune surveillance mechanisms required for tumor and viral control. This creates a fundamental design tension: grafts must be sufficiently protected to persist yet remain eliminable if malignant transformation or infection occurs. Incorporating inducible safety switches, such as inducible caspase-9, provides one practical safeguard. More broadly, immune evasion should be viewed as a tunable design parameter rather than an absolute state.

Despite rapid advances in multiplex genome editing and immune-instructive graft design, translation into clinical practice remains dominated by encapsulation devices and immunosuppression-supported grafts (28). This gap reflects practical constraints, including regulatory caution surrounding extensive genome editing, manufacturing challenges in ensuring product consistency, uncertainties regarding long-term genomic stability, and limited safety data for persistent checkpoint ligand expression. Encapsulation platforms prioritize retrievability and safety but remain constrained by fibrosis, hypoxia, and incomplete immune isolation.

Looking ahead, durable β-cell replacement will likely require convergence rather than competition among strategies. Optimized β-cell differentiation, rational immune-evasive engineering, immune-supportive local niches, and selectively applied systemic immune reprogramming will likely need to operate in concert. Together, these approaches reflect the biological reality that no single intervention can fully resolve the immune barriers inherent to T1D, and that durable graft function will depend on coordinated, context-aware immunomodulation rather than reliance on any single solution.
